# Automatic quantification of tenosynovitis on MRI of the wrist in patients with early arthritis: a feasibility study

**DOI:** 10.1007/s00330-018-5807-2

**Published:** 2018-11-12

**Authors:** Evgeni Aizenberg, Denis P. Shamonin, Monique Reijnierse, Annette H. M. van der Helm-van Mil, Berend C. Stoel

**Affiliations:** 1 Department of Radiology, Division of Image Processing, Leiden University Medical Center, P.O. Box 9600, 2300 RC Leiden, The Netherlands; 2 Department of Radiology, Leiden University Medical Center, Leiden, The Netherlands; 3 Department of Rheumatology, Leiden University Medical Center, Leiden, The Netherlands; 4 Department of Rheumatology, Erasmus Medical Center, Rotterdam, The Netherlands

**Keywords:** Rheumatoid arthritis, Tenosynovitis, Inflammation, Magnetic resonance imaging

## Abstract

**Objectives:**

Tenosynovitis (inflammation of the synovial lining of the sheath surrounding tendons) is frequently observed on MRI of early arthritis patients. Since visual assessment of tenosynovitis is a laborious task, we investigated the feasibility of automatic quantification of tenosynovitis on MRI of the wrist in a large cohort of early arthritis patients.

**Methods:**

For 563 consecutive early arthritis patients (clinically confirmed arthritis ≥ 1 joint, symptoms < 2 years), MR scans of the wrist were processed in three automatic stages. First, super-resolution reconstruction was applied to fuse coronal and axial scans into a single high-resolution three-dimensional image. Next, 10 extensor/flexor tendon regions were segmented using atlas-based segmentation and marker-based watershed. A measurement region of interest (ROI) was defined around the tendons. Finally, tenosynovitis was quantified by identifying image intensity values associated with tenosynovial inflammation using fuzzy clustering and measuring the fraction of voxels with these characteristic intensities within the measurement ROI. A subset of 60 patients was used for training and the remaining 503 patients for validation. Correlation between quantitative measurements and visual scores was assessed through Pearson correlation coefficient.

**Results:**

Pearson correlation between quantitative measurements and visual scores across 503 patients was *r* = 0.90, *p* < 0.001. False detections due to blood vessels and synovitis present within the measurement ROI contributed to a median offset from zero equivalent to 13.8% of the largest measurement value.

**Conclusion:**

Quantitative measurement of tenosynovitis on MRI of the wrist is feasible and largely consistent with visual scores. Further improvements in segmentation and exclusion of false detections are warranted.

**Key Points:**

• *Automatic measurement of tenosynovitis on MRI of the wrist is feasible and largely consistent with visual scores.*

• *Blood vessels and synovitis in the vicinity of evaluated tendons can contribute to false detections in automatic measurements.*

• *Further improvements in segmentation and exclusion of false detections are important directions of future work on the path to a robust quantification framework.*

## Introduction

Initiation of treatment in the early stages of rheumatoid arthritis (RA) has been associated with higher chances of drug-free sustained remission and improved quality of life [1]. Therefore, it is important to recognize patients who are at risk of progressing to RA as early as possible, either in the symptomatic phase of arthralgia, which precedes clinical arthritis, or in the earliest phases of clinically detectable arthritis. Recent studies suggest that MRI-detected inflammation can aid this task [2–4], especially in combination with serological markers [2]. Among the different types of inflammation observed on MRI of hands and wrists, it has been shown that tenosynovitis (inflammation of the synovial lining of the sheath surrounding tendons) is independently predictive of RA development, both in patients presenting with early arthritis and with arthralgia [2–5]. In addition, changes in MRI-detected tenosynovitis may be of interest in treatment response evaluation.

Assessment of tenosynovitis on MRI is commonly done according to the scoring method of Haavardsholm et al [6], in which a reader examines multiple tendon regions and estimates the thickness of peritendinous effusion or synovial proliferation with contrast enhancement. This is a laborious task, which requires the availability of trained, experienced readers. Automating the evaluation of tenosynovitis could offer standardized, high precision measurements derived directly from the image data and alleviate the time burden and cost associated with visual scoring. To date, limited research is available on this topic. Bowes et al have published a conference abstract on quantifying change in tenosynovitis over time in 34 RA patients receiving treatment [7], but data on single time point validation of these quantitative measurements with respect to visual scores are not publicly available.

In a recent study, we developed an automatic framework for measuring bone marrow edema (a strong predictor of radiographic progression in RA patients [8]) on MR images of the wrist [9]. In the work presented here, we sought to extend that framework to measure tenosynovitis of the extensor and flexor tendons of the wrist. Our aim was to investigate the feasibility of tenosynovitis quantification and assess the correlation between quantitative measurements and visual scores in a large cohort of early arthritis patients.

## Materials and methods

### Patients

A total of 563 early arthritis patients consecutively included in the Leiden Early Arthritis Clinic cohort [10] were studied. Mean age (±SD) was 54.9 (± 15.4) years; 350 patients (62.2%) were female. Inclusion required clinically confirmed arthritis by physical examination in ≥ 1 joints and symptom duration < 2 years. The cohort study was approved by the medical ethics committee of Leiden University Medical Center (Leiden, The Netherlands). All participants provided written informed consent.

### MRI scanning and visual scoring

**Fig. 1:**
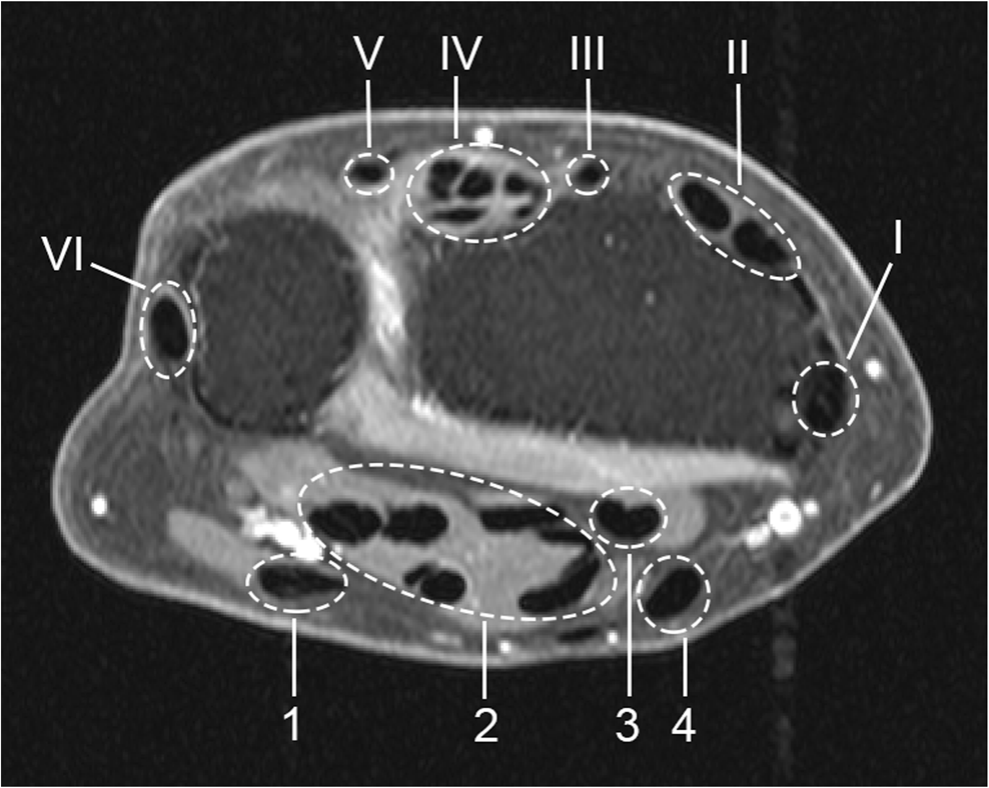
Tendon regions (compartments) scored for tenosynovitis, shown on axial MR image of the wrist (T1, post-gadolinium, fat-saturated). The six defined extensor compartments contain abductor pollicis longus, extensor pollicis brevis (I); extensor carpi radialis longus, extensor carpi radialis brevis (II); extensor pollicis longus (III); extensor digitorum communis, extensor indicus proprius (IV); extensor digiti quinti proprius (V); and extensor carpi ulnaris (VI). The four flexor regions contain flexor carpi ulnaris (1); ulnar bursa, including flexor digitorum profundus and superficialis tendon quartets (2); flexor pollicis longus (tendon) in radial bursa (3); and flexor carpi radialis (4). Note: the flexor carpi ulnaris does not have a tenosynovial sheath; nevertheless, inflammation around this tendon is also observed, and therefore, enhancement of tissue surrounding this tendon is scored [11]

The wrist joint of the most painful side (or the dominant side in cases of equally severe symptoms on both sides) was scanned with a 1.5T extremity MR scanner (GE Healthcare) using a 100-mm coil, with contrast enhancement and frequency-selective fat saturation (T1-Gd). Table 1 summarizes the acquisition parameters. In line with the definitions proposed by Haavardsholm et al [6], tenosynovitis was evaluated in six extensor compartments and four flexor regions within the wrist joint (Fig. 1). Visual scoring was independently performed by two trained readers blinded to clinical data. For each anatomical region, the readers provided a grade on a 0–3 scale based on the estimated maximum width of peritendinous effusion or synovial proliferation with contrast enhancement, as follows: grade 0, normal; grade 1, < 2 mm; grade 2, ≥ 2 mm and < 5 mm; grade 3, ≥ 5 mm. The scoring region was bounded by the distal radius/ulna proximally and the hook of the hamate distally. The intra-reader intra-class correlation coefficients (ICCs) of the two readers for the total tenosynovitis score (sum across all tendon regions), based on 40 MRIs scored twice, were 0.99 and 0.83. The inter-reader ICC for the total tenosynovitis score, based on all 563 MRIs, was 0.87. In what follows, the mean score of the two readers was always considered.

**Table 1:** MRI sequences

	Coronal scan	Axial scan
Repetition time (ms)	650	570
Echo time (ms)	17	7
Acquisition matrix	364 × 224	320 × 192
Echo train length	2	2
Slice thickness (mm)	2	3
Slice gap (mm)	0.2	0.3

Described are the acquisition parameters of T1-weighted fast spin-echo sequences with frequency-selective fat saturation obtained after intravenous injection of Gd-chelate (gadoteric acid, Guerbet, Paris, France, standard dose of 0.1 mmol/kg)

### Quantitative image analysis framework

#### Super-resolution reconstruction

The coronal and axial MR scans compensate each other in terms of anatomical detail, since the slice thickness in each of the scans (2 mm in coronal; 3 mm in axial) is much larger than the in-plane spacing between voxels (~ 0.2 mm). In order for a quantitative framework to make use of all available image data in a compact and efficient manner, it is desirable to fuse the two scans into a single 3D image using super-resolution reconstruction (SRR). The application of SRR to MR images of the wrist has been detailed in our previous work [9]. We applied the method of Poot et al [12] with Laplacian regularization (*λ* = 0.05).

#### Measurement region of interest

The computation of the ROI required automatically segmenting the tendons, carpal bones, distal radius/ulna, and the image region bounded by skin. The bones and initial landmarks for the tendon regions were obtained using atlas-based segmentation [13]. The atlas consisted of 13 early arthritis patients (separate dataset, excluding patients evaluated visually and quantitatively in this study). For each atlas patient, the tendon regions and bones were manually segmented in the axial T1-Gd images and then extended to SRR space by nearest neighbor interpolation. After spatially mapping every atlas image onto the target image using the Elastix toolbox [14–16], a majority vote was applied across all mappings, determining whether a voxel would be labeled as one of the tendons, bones, or neither. It should be noted that all atlas images contained the right wrist joint. For segmentation of the left wrist, atlas images were horizontally mirrored prior to registration.

**Fig. 2:**
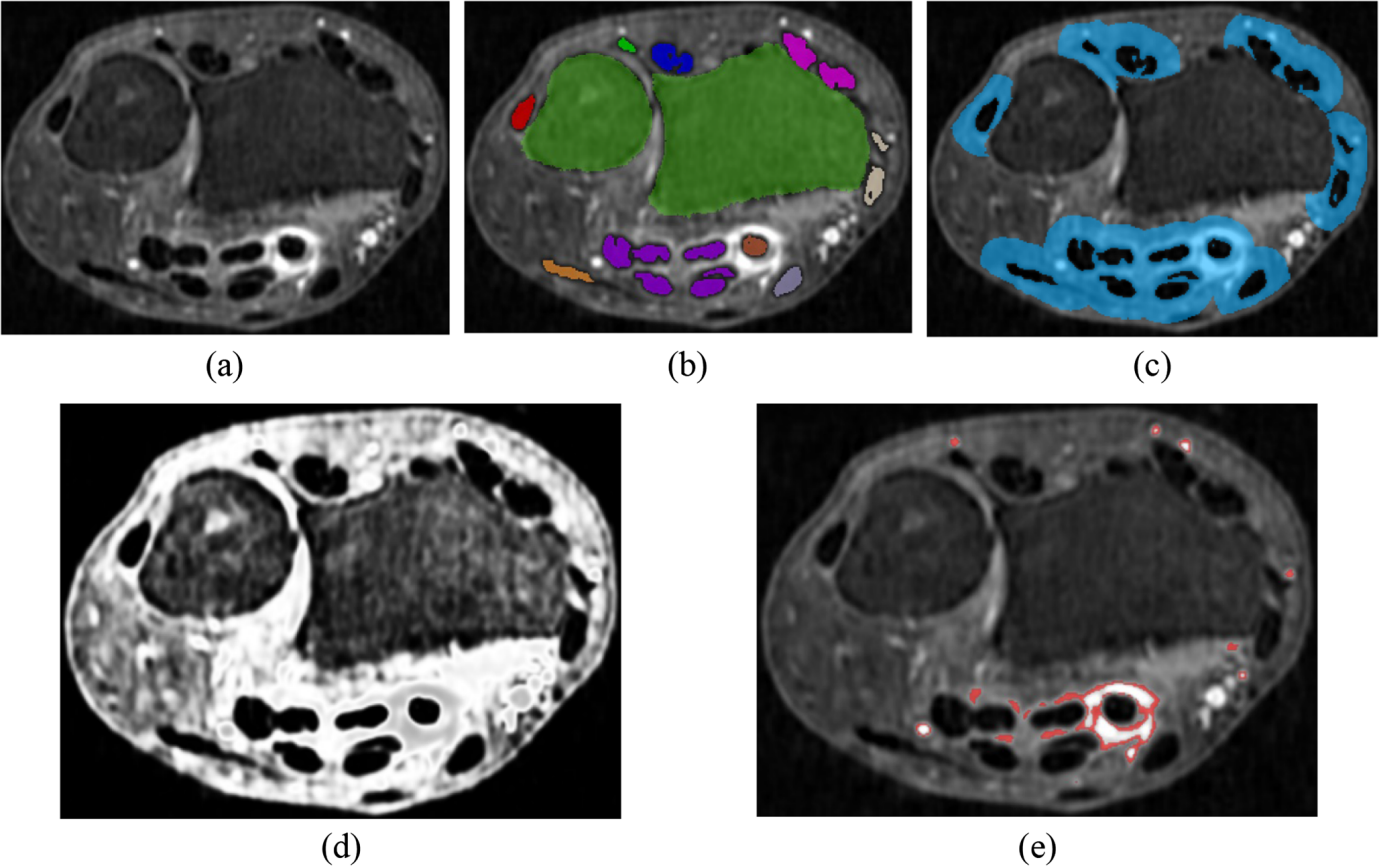
SRR image of the wrist (**a**), segmented tendon regions and bones (**b**), the resulting measurement ROI (**c**) in which tenosynovitis is quantified (*D* = 3 mm), C2 probability map (**d**), ROI locations included in the quantitative measurement marked in red (**e**). Image depicted in the figure received a total visual score of 1: grade 1 tenosynovitis in flexor region 3. Some of the voxels identified in neighboring flexor region 2 corresponded to a low grade enhancement, but were not picked up in visual scoring. Several blood vessels located within the ROI were also included, introducing a number of false detections that counted towards the quantitative measurement

Having obtained initial landmarks for the tendon regions, the tendons were segmented by a similar approach to Chen et al [17] using marker-based watershed segmentation [18–20], followed by removal of segmented regions whose intensity was > 75 (tendons are characterized by low image intensities on T1-Gd images) or whose volume was < 0.01 ml. An example of the resulting segmentations is shown in Fig. 2b.

In order to segment the image region bounded by skin, the entire image extent of the hand was approximated. First, the background was segmented by performing region growing with seeds placed at the four corners of each image slice. Then, the resulting binary image was inverted and the largest connected component was retained.

Finally, for each segmented tendon, a distance transform was performed and voxels within a fixed distance (*D*) of the tendons were included in the measurement ROI as long as these voxels were not part of other labeled structures. The distal radius/ulna boundary of the ROI was determined by identifying the axial slice where the two bones were closest to each other. The hook of the hamate boundary was determined by searching for the axial slice with the largest number of segmented hamate voxels. An example of the resulting measurement ROI is shown in Fig. 2c. As detailed in the optimization section, the value of distance parameter *D* was obtained by maximizing correlation with visual scores on a training set of patients.

#### Assessment of tendon segmentation accuracy

To assess the accuracy of tendon segmentation, a leave-one-out cross-validation was performed. In each of the 13 runs, 12 out of 13 atlas images would constitute the atlas set, and the remaining image would be used as the target image to be segmented. The result was validated against manual segmentation of the axial image. Segmentation accuracy was evaluated by computing precision and recall rates for each of the 10 tendon regions. Here, precision rate refers to the fraction of voxels segmented by the algorithm that overlap with the manual segmentation, while recall rate refers to the fraction of voxels within the manual segmentation that were correctly segmented by the algorithm.

#### Tenosynovitis quantification

Tenosynovitis is characterized by high signal intensity on T1-Gd (fat-suppressed) images due to contrast enhancement. Intensity values vary per acquisition, depending on the relative strength of contrast enhancement, the homogeneity of the fat suppression, and the inherent magnetic field inhomogeneities of the MR scanner. To account for these acquisition-specific intensity ranges of tenosynovitis, fuzzy C-means clustering [21, 22] was applied to the intensity values of all voxels in each image, assuming two clusters. This yields two probability map images, where each voxel contains the probability of that location belonging to the respective cluster. Let C2 be the cluster whose center value is the higher of the two computed cluster centers. As Fig. 2d illustrates, high probabilities (bright voxels) within the C2 probability map correspond to locations of healthy synovial tissue. Since our focus is on regions of inflammation, where image intensity is expected to be higher compared to healthy synovium, voxels whose intensity was lower than the value of C2 cluster center were removed, resulting in a one-sided C2 probability map.

Tenosynovitis was then quantified by computing the fraction of voxels within the measurement ROI whose one-sided C2 probability values *p*_*C*2_ were bounded by *T*_*L*_ ≤ *p*_*C*2_ < *T*_*H*_. As detailed below, the numeric values of the lower and upper thresholds (*T*_*L*_, *T*_*H*_) were optimized on a training set of patients to maximize correlation with visual scores.

### Optimization

In order to optimize the (*T*_*L*_, *T*_*H*_) thresholds and distance parameter *D* based on correlation with visual scores, a training set of patients was defined. The number of patients with low tenosynovitis (grades 0 and 1) in our early arthritis cohort was much larger than the number of patients with moderate-severe tenosynovitis (grades 2 and 3). Therefore, a random sampling of the cohort would not guarantee inclusion of patients with severe tenosynovitis in the training sample. In order to produce a more balanced training set representing the full range of tenosynovitis severity, we used a similar sampling approach as in our study on bone marrow edema [9]. We categorized 563 patients by the maximum visual score (*V*_max_) across the scored tendon regions. Three sampling categories were defined corresponding to three severity intervals within *V*_max_ range (0–3): *V*_max_ = 0, 0 < *V*_max_ ≤ 1, 1 < *V*_max_ ≤ 3. Table 2 lists the defined categories and the number of patients that fall into each category. Next, 20 patients were randomly selected from each category to form a training set of 60 patients. The optimal distance and threshold values were found by computing the quantitative measurement for *D* = 1, 2, 3, 4, 5, and 6 mm and all possible combinations (step size 0.01) of (*T*_*L*_, *T*_*H*_) and determining which set of parameters maximized the Pearson correlation coefficient *r* between the total visual score of tenosynovitis (sum across all tendon regions) and the total quantitative tenosynovitis measurement.

**Table 2:** Training set sampling categories

Severity category index	*V*_max_interval	Number of patients
0	*V*_max_ = 0	200
1	0 < *V*_max_ ≤ 1	261
2	1 < *V*_max_ ≤ 3	102

Note: Random sampling across all categories would form a training set that mainly consists of patients with *V*_max_ ≤ 1. In contrast, randomly selecting 20 patients from category 2, for example, guarantees that the training set will include 20 patients in which at least one tendon region received a visual score greater than 1. Thus, random sampling from each severity category helps ensure *D*, *T*_*L*_, and *T*_*H*_ are optimized with respect to the entire range of tenosynovitis severity

### Validation

After optimizing and locking the values of *D*, *T*_*L*_, and *T*_*H*_, the method was validated by computing the quantitative tenosynovitis measurement for the 503 patients that were not part of the training set and evaluating the Pearson correlation coefficient between the total visual score and the total quantitative measurement.

### Statistical analysis

When assessing the Pearson correlation coefficient between visual scores and quantitative measurements, *p* values below 0.05 were considered to be statistically significant. Statistics were computed using MATLAB R2015b (The MathWorks, Inc.).

## Results

### Assessment of tendon segmentation accuracy

**Fig. 3:**
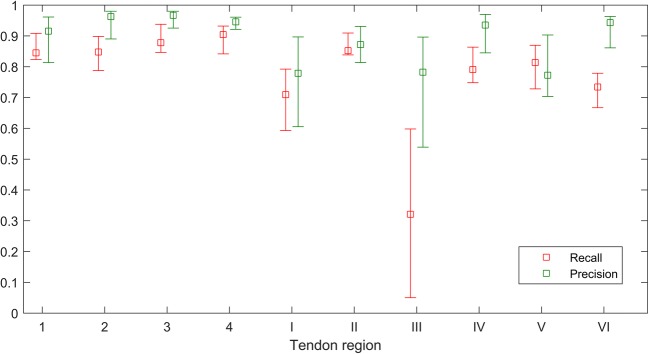
Median and interquartile range of recall and precision rates of tendon region segmentation across 13 atlas images

The median and interquartile range (IQR) of recall and precision rates of tendon region segmentation across 13 atlas images are shown in Fig. 3. Flexor regions exhibited high precision rates (median values ranging from 0.92 to 0.97) and moderate-high recall rates (median values ranging from 0.85 to 0.90). The rates were generally lower for extensor regions and exhibited more variability (median precision ranging from 0.78 to 0.94 and median recall ranging from 0.32 to 0.85). The lowest recall (including three failed segmentations) was observed for extensor region III.

### Optimization

**Fig. 4:**
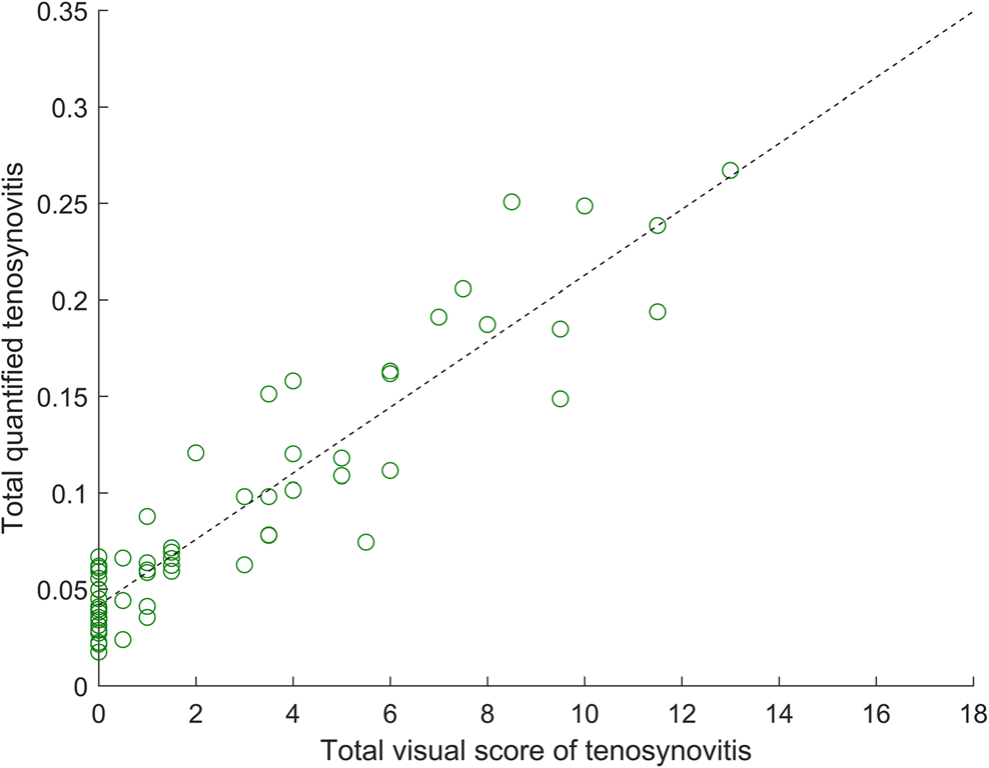
Scatter plot of total quantitative measurements of tenosynovitis versus total visual scores for 60 training set patients. Each data point represents a single patient. Pearson correlation *r* = 0.93, *p* < 0.001 (*D* = 3 mm, *T*_*L*_ = 0.82, *T*_*H*_ = 0.94). Dashed black line represents linear regression fit

The highest Pearson correlation value (*r* = 0.93, *p* < 0.001) between the total visual score of tenosynovitis and the total quantitative measurement over 60 training set patients was observed with distance parameter *D* = 3 mm and threshold values *T*_*L*_ = 0.82 and *T*_*H*_ = 0.94. As illustrated by the scatter plot in Fig. 4, increasing levels of tenosynovitis severity were fairly consistently matched with increasing values of the quantitative measurement. The measurements of patients with total visual score 0 had a median offset from zero of 0.04 (IQR 0.03–0.05), constituting 14.8% of the largest observed value of 0.27 for the most severely affected patients. Figure 2e shows an example of measurement ROI locations that were counted towards the quantitative measurement.

### Validation

**Fig. 5:**
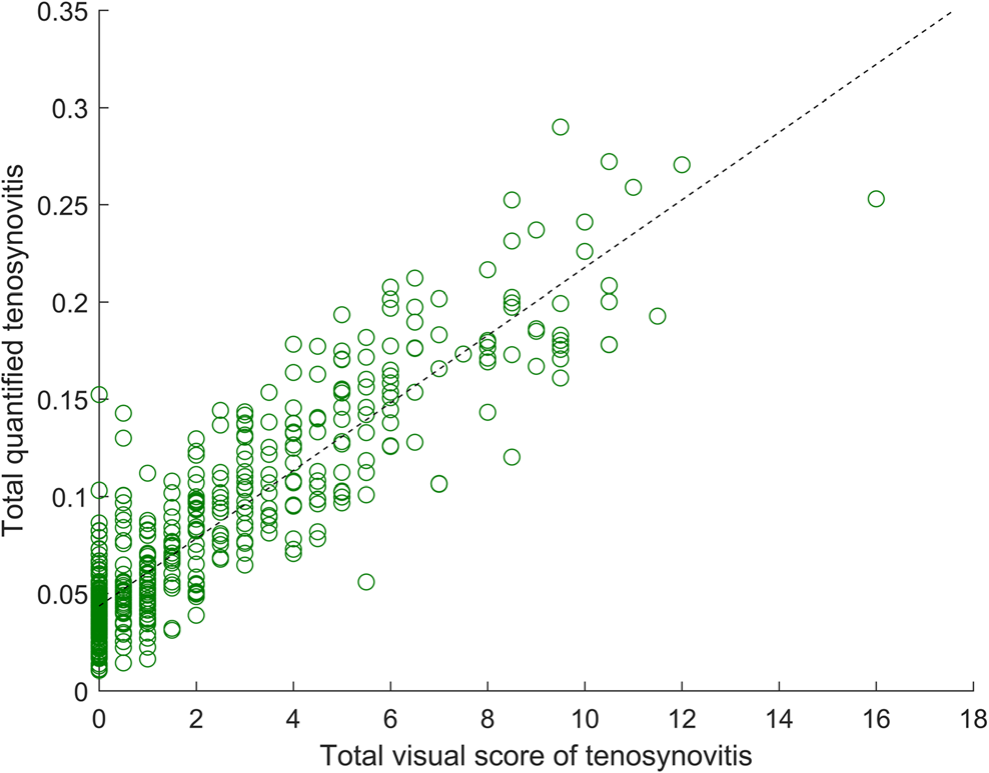
Scatter plot of total quantitative measurements of tenosynovitis versus total visual scores for 503 validation set patients. Each data point represents a single patient. Pearson correlation *r* = 0.90, *p* < 0.001 (*D* = 3 mm, *T*_*L*_ = 0.82, *T*_*H*_ = 0.94). Dashed black line represents linear regression fit

Having obtained the optimized parameter values, the quantitative measurement was computed for 503 patients, and correlation was assessed. The resulting Pearson correlation coefficient was *r* = 0.90 and *p* < 0.001. The scatter plot in Fig. 5 shows that majority of patients exhibited a consistent trend of increasing quantitative measurements with increasing levels of tenosynovitis severity. The measurements of patients with total visual score 0 had a median offset from zero of 0.04 (IQR 0.03–0.05) (same as in training), constituting 13.8% of the largest observed value of 0.29. Visual inspection of results indicated that blood vessels and synovitis present within the measurement ROI were often mistakenly counted as tenosynovitis by the quantitative measurement, increasing its numeric value. The strongly outlying case of a patient with visual score 0 and a quantitative measurement of 0.15 was caused by a failed tendon segmentation due to an unusually low intensity distribution of healthy synovium.

## Discussion

In this study, we investigated the feasibility of automatic quantification of tenosynovitis on MRI of the wrist in a large cohort of early arthritis patients. The presented method extended our previously developed atlas-based framework [9] to the extensor and flexor tendons of the wrist, providing the landmarks necessary for tendon segmentation and definition of the ROI in which tenosynovitis was measured. The results exhibited strong correlation between quantitative measurements and visual scores. Quantitative measurements should not be viewed as a replication of visual scoring and therefore this study assessed consistency and correlation, rather than absolute agreement. The observed correlation is especially encouraging considering that there is an inherent degree of variability within visual scores due to the interval-based definition of the visual grades. These findings indicate that automatic quantification of tenosynovitis on MRI of early arthritis patients is feasible, and that quantitative measurements are largely consistent with visual scoring. However, this study also brings out multiple challenges pertinent to the quantification task, such as moderate segmentation performance and sources of false detections. As detailed in the following discussion, these are important issues that will need to be addressed on the path to a robust quantification framework.

Interestingly, the overall moderate tendon segmentation recall rates did not seem to have a strong adverse effect on correlation between quantitative measurements and visual scores. This can be explained by the fact that even if a tendon is partially segmented, the measurement ROI around the segmentation is still likely to include the tendon’s synovial lining. Although the ROI will then also include voxels inside the tendon, on T1-Gd images tendons are characterized by low image intensities which do not contribute towards the inflammation measurement; one exception is enhancement due to concomitant tendinitis. It should be recognized, however, that in this study, we measured the total inflammation across all evaluated tendon regions, which may have reduced sensitivity to errors made on the individual region level. This is particularly relevant when considering the low recall rates for extensor region III. The 3/13 failed cross-validation cases indicate that reliable quantification of inflammation around this tendon was not always feasible. A likely reason for this is that extensor region III is the smallest of the 10 tendon regions and exhibits higher curvature, making the placement of atlas-based landmarks more challenging. One type of segmentation error that had no effect on total measurements was mislabeling of one tendon region as another; however, future studies must thoroughly assess mislabeling errors if evaluation of tenosynovitis on individual region level is of interest. More generally, it should be noted that inaccuracies in tendon segmentation do affect the total number of voxels included in the measurement ROI and thereby introduce some variability in the quantitative measurement. Therefore, improving segmentation accuracy is an important direction of future work both for measurement precision and accurate evaluation of tenosynovitis on the individual region level.

As illustrated by Fig. 2e, locations counted towards the quantitative measurement did not always include all voxels within the inflammation, but most voxels along the boundary of the inflammation were typically included. One possible reason for this is that the threshold parameters (*T*_*L*_, *T*_*H*_) were optimized with respect to scores that reflect the maximum thickness of peritendinous effusion or synovial proliferation in each tendon region. Maximum thickness is not equivalent to total volume, and therefore, it is plausible that some voxels within the inflammation were not included in the measurement. Figure 2e also illustrates that one drawback of the current method is that blood vessels introduce false detections that contribute towards the quantitative measurement. This observation explains one of the factors behind the consistent offset from zero both during training and validation. Future improvements should include detection of blood vessels and their exclusion from the measurement ROI.

Visual inspection of quantification results indicated that synovitis present within the measurement ROI (for example, between carpal bones and tendons) was mistakenly counted as tenosynovitis by the quantitative measurement. In visual scoring, trained readers employ their expertise and pattern recognition to classify the observed inflammation as either synovitis or tenosynovitis. The presented method did not include such classification, and therefore, it is not surprising that it counted all inflammation detected within the measurement ROI as tenosynovitis. This is another contributing factor to the offset observed in training and validation. Since synovitis is often present in joints in the vicinity of tendons affected by tenosynovitis [11], a more specific definition of the measurement ROI is warranted.

In conclusion, the presented method provides a reference on the path to automatic quantification of tenosynovitis on MRI and lays out possible directions for future improvements. The common presence of tenosynovitis in RA and its association with RA development in arthralgia and early arthritis patients motivate the development of quantitative measurement techniques. These advances would aid clinical researchers by standardizing interpretation and allowing them to dedicate more resources to analysis rather than visual scoring, facilitating both research and potential clinical implementation.

## Abbreviations

ICC: Intra-class correlation coefficient
IQR: Interquartile range
RA: Rheumatoid arthritis
SD: Standard deviation
SRR: Super-resolution reconstruction
T1-Gd: T1-weighted fat-saturated sequence with contrast enhancement

## References

[1] Ajeganova S, Huizinga T (2017). Sustained remission in rheumatoid arthritis: latest evidence and clinical considerations. Ther Adv Musculoskelet Dis.

[2] van Steenbergen HW, Mangnus L, Reijnierse M (2016). Clinical factors, anticitrullinated peptide antibodies and MRI-detected subclinical inflammation in relation to progression from clinically suspect arthralgia to arthritis. Ann Rheum Dis.

[3] Kleyer A, Krieter M, Oliveira I (2016). High prevalence of tenosynovial inflammation before onset of rheumatoid arthritis and its link to progression to RA-A combined MRI/CT study. Semin Arthritis Rheum.

[4] Nieuwenhuis WP, van Steenbergen HW, Mangnus L et al (2017) Evaluation of the diagnostic accuracy of hand and foot MRI for early rheumatoid arthritis. Rheumatology (Oxford) 56:1367–1377. 10.1093/rheumatology/kex167 10.1093/rheumatology/kex167 28460018

[5] Aizenberg E, ten Brinck RM, Reijnierse M, et al (2018) Identifying MRI-detected inflammatory features specific for rheumatoid arthritis: two-fold feature reduction maintains predictive accuracy in clinically suspect arthralgia patients. Semin Arthritis Rheum 10.1016/j.semarthrit.2018.04.005 10.1016/j.semarthrit.2018.04.005 PMC7615878 29853189

[6] Haavardsholm EA, Østergaard M, Ejbjerg BJ (2007). Introduction of a novel magnetic resonance imaging tenosynovitis score for rheumatoid arthritis: reliability in a multireader longitudinal study. Ann Rheum Dis.

[7] Bowes MA, Guillard G, Vincent GR, et al (2015) Quantitative MRI measurement of tenosynovitis demonstrates differing responses of synovitis and tenosynovitis after RA treatment. In: 2015 ACR/ARHP Annual Meeting

[8] Hetland ML, Ejbjerg B, Hørslev-Petersen K (2009). MRI bone oedema is the strongest predictor of subsequent radiographic progression in early rheumatoid arthritis. Results from a 2-year randomised controlled trial (CIMESTRA). Ann Rheum Dis.

[9] Aizenberg E, Roex EAH, Nieuwenhuis WP (2018). Automatic quantification of bone marrow edema on MRI of the wrist in patients with early arthritis: a feasibility study. Magn Reson Med.

[10] de Rooy DPC, van der Linden MPM, Knevel R (2011). Predicting arthritis outcomes--what can be learned from the Leiden Early Arthritis Clinic?. Rheumatology (Oxford).

[11] Nieuwenhuis WP, Krabben A, Stomp W (2015). Evaluation of magnetic resonance imaging-detected tenosynovitis in the hand and wrist in early arthritis. Arthritis Rheumatol.

[12] Poot DHJ, Van Meir V, Sijbers J (2010). General and efficient super-resolution method for multi-slice MRI. Med Image Comput Comput Assist Interv.

[13] Rohlfing T, Brandt R, Menzel R, et al (2005) Quo vadis, atlas-based segmentation? In: The Handbook of Medical Image Analysis - Volume III: Registration Models. pp 435–486

[14] Klein S, Staring M, Murphy K (2010). elastix: a toolbox for intensity-based medical image registration. IEEE Trans Med Imaging.

[15] Shamonin DP, Bron EE, Lelieveldt BPF (2013). Fast parallel image registration on CPU and GPU for diagnostic classification of Alzheimer’s disease. Front Neuroinform.

[16] Aizenberg E, Shamonin DP (2018) Elastix parameters for atlas-based segmentation of bones and tendons on MR images of the wrist. http://elastix.bigr.nl/wiki/index.php/Par0051

[17] Chen H-C, Wang Y-Y, Lin C-H (2013). A knowledge-based approach for carpal tunnel segmentation from magnetic resonance images. J Digit Imaging.

[18] Vincent L, Soille P (1991). Watersheds in digital spaces: an efficient algorithm based on immersion simulations. IEEE Trans Pattern Anal Mach Intell.

[19] Vincent L (1993). Morphological grayscale reconstruction in image analysis: applications and efficient algorithms. IEEE Trans Image Process.

[20] Xu S, Liu H, Song E (2011). Marker-controlled watershed for lesion segmentation in mammograms. J Digit Imaging.

[21] Bezdek JC (1981) Pattern recognition with fuzzy objective function algorithms

[22] Amiri M (2003) Yashil’s Fuzzy C-Means Clustering MATLAB Toolbox Ver 1.0. http://ce.sharif.edu/~m_amiri/project/yfcmc/index.htm

